# The Decline of Paget’s Disease of Bone and Domestic Coal Use—A Hypothesis

**DOI:** 10.1007/s00223-024-01241-0

**Published:** 2024-06-20

**Authors:** Tim Cundy

**Affiliations:** Faculty of Medical & Health Sciences, University of Auckland, Auckland, Aotearoa New Zealand

**Keywords:** Paget’s disease of bone, Epidemiology, Coal, Death certification, History

## Abstract

The cause of Paget’s disease of bone (PDB) is unknown. It emerged as a distinct entity in Britain in the late nineteenth century when it was prevalent, and florid presentation not uncommon. Epidemiological surveys in the 1970s showed that Britain had a substantially higher prevalence of PDB than any other country. Studies in the late twentieth and early twenty-first centuries have documented an unexplained change in presentation, with a greatly reduced prevalence and less severe disease than formerly. The emergence of PDB in Britain coincided with rapid industrialization which, in turn, was driven by the use of coal for energy. In the home, bituminous coal was customarily burnt on an open hearth for heating. Using data on coal production, population size, and estimates of domestic use, the estimated exposure to domestic coal burning rose threefold in Britain during the nineteenth century and began to fall after 1900. This pattern fits well with the decline in PDB documented from death certification and prevalence surveys. Colonists moving from Britain to North America, Australia and New Zealand established coal mines and also used coal for domestic heating. PDB was found in these settler populations, but was largely absent from people indigenous to these lands. In all parts of the world PDB prevalence has fallen as the burning of coal in open hearths for domestic heating has reduced. The nature of the putative factor in coal that could initiate PDB is unknown, but possible candidates include both organic and inorganic constituents of bituminous coal.

## Introduction

**Fig. 1:**
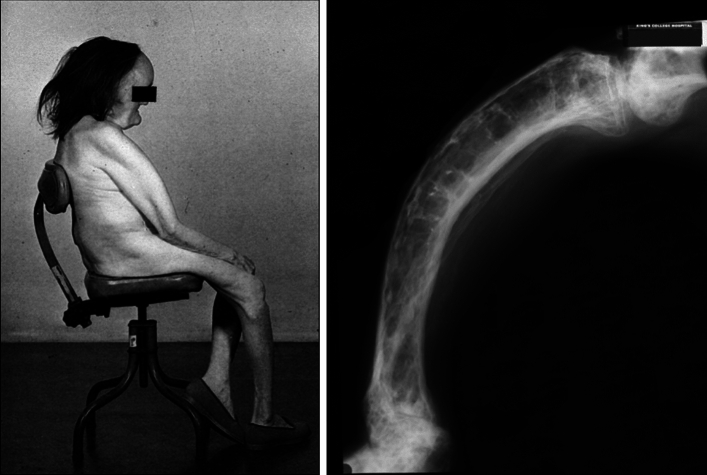
Two people with Paget’s disease seen by the author in London in the mid-1970s. Left: severe polyostotic involvement affecting skull and humerus. Right: severe bowing deformity of the tibia. Presentations with such severe disease are now exceptionally rare

Paget’s disease of bone (PDB) is an unusual condition not least because highly effective therapy has preceded a full understanding of its etiology and biology. The characteristic accelerated turnover in affected bones can be effectively suppressed, and symptoms relieved, by bisphosphonate treatment, but how and why PDB develops remains an enigma. Research over the last two decades has demonstrated definite associations with mutation in a number of genes, most commonly *SQSTM1* [1, 2], but genetic predisposition cannot explain its changing epidemiology. In all parts of the world where PDB was formerly prevalent, studies in recent decades have shown not only a reduction in prevalence, but also amelioration in its presentation (diagnosis at a greater age and with less extensive disease than formerly) [3–5]. When I was a junior doctor in London in the mid-1970s elderly people with florid disease were still to be encountered (Fig. 1), but over the last 50 years such presentations have almost completely disappeared. This change in epidemiology is clearly at odds with the notion that PDB is primarily a genetic disorder [1, 2], particularly as the same amelioration of phenotype is evident in offspring inheriting *SQSTM1* mutations from their clinically affected parents [6, 7]. There must therefore be (or have been) an important environmental influence at play. The putative environmental factor has not been identified, perhaps unsurprisingly since it seems no longer to be present. An alternative approach to understanding the phenomenon is to examine conditions extant when and where PDB emerged as a distinct clinical identity, how those conditions have changed, and whether the time course of that change matches that in the epidemiology of PDB.

## The History of Paget’s Disease

PDB is identifiable in ancient skeletons, and its origins lie in Western Europe [8]. The oldest skeletons date from the Roman era, and the great majority are from England, with only a handful of cases from Germany, France and Portugal. The number of skeletons found to be affected increased from the late medieval period (from 1066 CE) onwards [8]. Excavations from a large burial site in North East England estimated the prevalence of the disease was 1.7% in skeletons interred between 900 and 1500 CE and 3.1% in those interred between 1500 and 1850 CE [9].

James Paget practiced in London from 1834 and published his first paper on his now eponymous disease in 1876. He detailed the case a man from ‘a cold and damp place in the North of England’ who at the age of 46 began to experience bone pain and noted bowing of his left tibia. He consulted Paget two years later in 1856. Paget noted the deformity of the tibia and also suspected there was deformity in the left femur. Over the next 19 years the man’s deformities, which included skull enlargement, progressed and he died of an osteosarcoma in 1876. In the same paper, Paget described four other cases all presenting in the 1860s [10]. In a second paper, published in 1882 Paget described seven additional cases, and also acknowledged that individual cases described in the German literature from Bohemia (1867) and Freiburg (1873) were probably the same disease [11]. It is worth noting that compared to contemporary cases, the 11 people Paget described were young, with the mean age at the onset of symptoms only 52 years. Paget was a very astute clinician, but with no biochemistry or radiology to assist diagnosis, even he would have been able to recognise only those with the most severe clinical manifestations, so one can be reasonably certain that there would have been many unrecognized cases at that time. Death certification data from people born in that era (discussed below) also indicates that the disease was prevalent.

To summarize: the disease has been identified in a few archaeological skeletons dating from the Roman era but over subsequent centuries it seems to have become more common in Britain. By the mid-nineteenth century, again in Britain, the disorder had become clinically evident, presenting in florid form in people in their fifties, and was presumably quite prevalent.

## The Changing Epidemiology

**Fig. 2:**
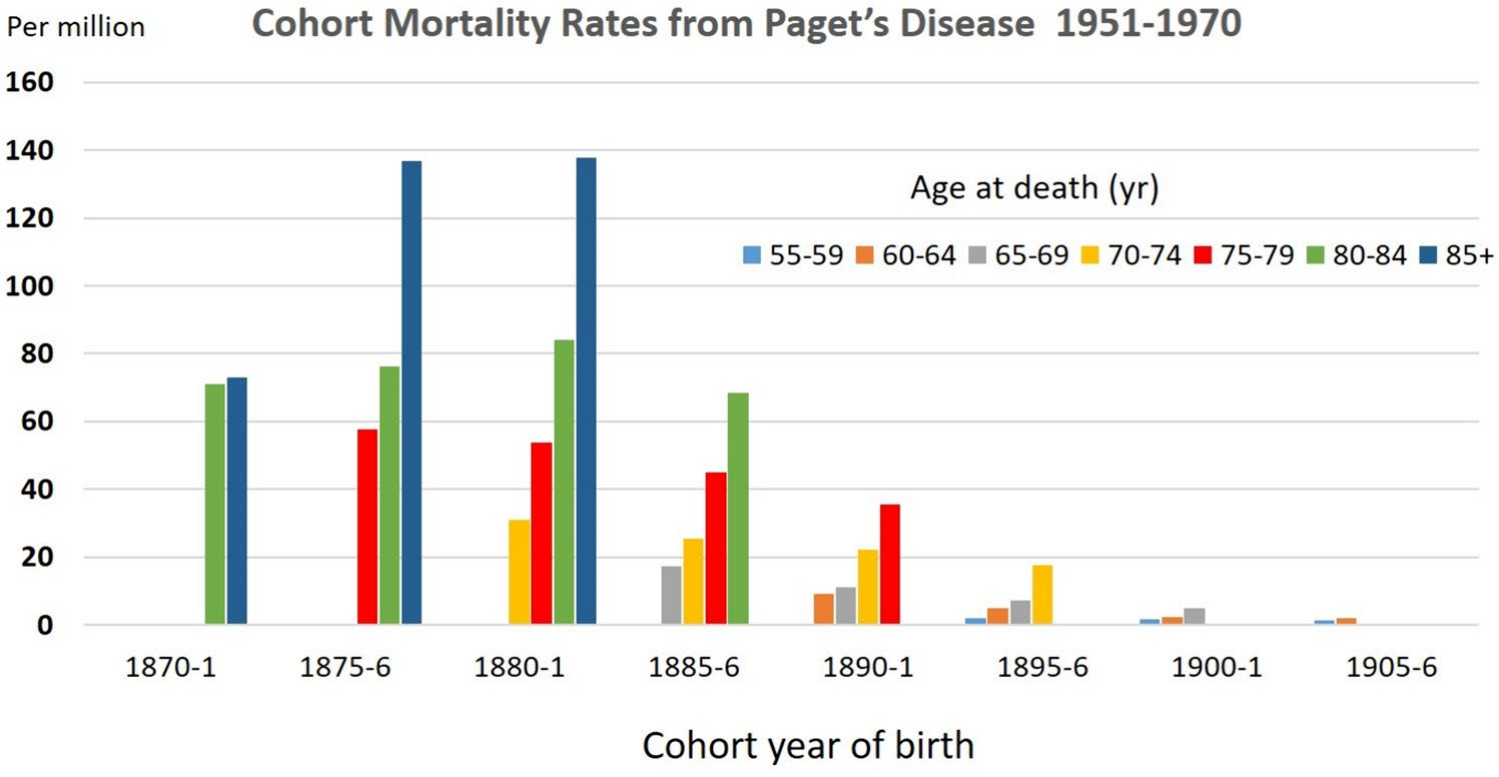
The proportion of death certificates for men in England and Wales in the period 1951–70 that mentioned Paget’s disease of bone, according to year of birth and age at death. Note the marked decline in mortality in cohorts born after 1880–1. For example, at age 75–79 mortality rates were 39% lower in the cohort born 1890–1 than in the cohort born 1875–76. Data for women (not shown) demonstrated lower rates than men but an identical temporal trend. Figure redrawn from the data of Barker and Gardner [12]

In 1974, Gardner and Barker were the first to suggest that clinical features of PDB had begun to change. Utilising data from hospital discharges and death certification from people born in the late nineteenth century they showed that within Britain not only were there regional variations in hospital discharge rates for Paget’s disease (being lower in South East England and some rural areas of Scotland), but also that successive cohorts born after 1880–1801 showed a progressive fall in mortality attributed to PDB [12] (Fig. 2). In a subsequent paper they noted parallel changes in PDB-associated mortality in the USA, and progressive reductions in mortality from primary bone tumors in adulthood, in both the USA and England & Wales—such tumors most commonly being osteosarcoma arising in pagetic bone [13].

Radiological surveys to estimate the prevalence of Paget’s disease were undertaken in many countries from the 1970s onwards. In such surveys a large number of sequential radiographs of the pelvis, taken for any reason, were examined for the characteristic signs of the disease. In ~ 70% of affected people the disease involves one or more of the lumbar spine, sacrum, pelvis or proximal femur, so is visible on such a radiograph. The surveys showed not only marked differences in prevalence between countries, but also regional differences within countries. The United Kingdom had the highest country prevalence (5.5% of people over 55 years), particularly in towns in the cold and damp north-west of England where rates > 7% were seen [14, 15]. Further studies in European cities showed wide variation in prevalence, but in no country was the prevalence as high as in Britain. The highest rates were in French towns (2.0–2.7%), with intermediate rates in Germany (1.1–1.3%) and Ireland (0.7–1.7%), and the lowest in Scandinavia (0.4–0.5%) [16, 17].

In several countries repeat radiographic surveys were undertaken about twenty years after the first, and in almost all areas the proportion of the population affected had fallen by at least half [3–5]. Over the same period there had also been a remarkable reduction in the severity of the disease, as assessed by the proportion of the skeleton involved on bone scintigraphy [18]. The great majority of contemporary patients—even *SQSTM1* mutation carriers—now have only 1 or 2 bones involved and are asymptomatic [19]. Severe polyostotic disease, of the type recognized by Paget and illustrated in Fig. 1, is now a rarity. The average age at diagnosis has been steadily increasing, so the change cannot be attributed to diagnosis earlier in life and medical intervention [18]. The disappearance of Paget’s disease continues apace: in a large survey of general practice data in the UK the incidence of clinically diagnosed Paget’s disease decreased by > 60% between 1999 and 2015 [20]. In Lancaster in the north-west of England the prevalence of PDB has fallen by an order of magnitude—from 8 to 0.8%—in 40 years [21]. In the ZiPP study of *SQSTM1* mutation carriers, the rate at which PDB had developed in the placebo group by the end of the study (2021) was only one eighth that anticipated when the study was registered 13 years earlier [22]. So there is little question that the nature of Paget’s disease has changed from its apparent peak in the late nineteenth and early twentieth century. But how can we explain its geographical distribution centred on Britain, and its remarkable temporal trajectory?

## What Type of Environmental Factor?

Barker and Gardner tentatively suggested that improved vitamin D nutrition in childhood might be relevant [12] but this seems unlikely. Paget’s disease is not a consequence of severe vitamin D deficiency, and vitamin D deficiency is seen all around the world. Following the suggestion that the inclusion bodies seen by electron microscopy in pagetic osteoclasts resembled viral particles [23] searches for environmental factors became focussed on potential infectious agents. Candidates that were explored include canine distemper and measles, but the epidemiology of these diseases does not fit that of Paget’s disease—both these viral infections occur worldwide. Immunization (of dogs) against distemper virus began in the late 1950s and immunization (of humans) against measles began around 1969—too late to explain the historical decline in prevalence of Paget’s disease. The inclusion bodies, which are not unique to Paget’s disease, but probably relevant to its pathogenesis, are now thought to be aggregates containing ubiquitin and RNA-binding proteins, such as, hnRNPA1, and hnRNPA2B1, but may also include proteins that mediate ubiquitin-dependent autophagy, including p62/SQSTM1, and VCP amongst others, that form because of defects in the cellular degradation machinery [1, 24].

Could the environmental factor be a pollutant? Lever suggested that calcium arsenate toxicity related to cotton processing in Lancashire mill towns might be significant in the high prevalence focus in north-west England. Cotton was imported to the mills of Lancashire from the southern states of America. To combat boll weevil infestation, calcium arsenate was used on cotton plants as an insecticide between 1923 and 1945 [25]. Lever argued that the cessation of calcium arsenate use could have contributed to the decline in prevalence between the 1970s and 1990s surveys, but it cannot explain the decline in prevalence seen in other parts of Britain or the rest of the world.

## Possible Environmental Factors that Changed in Britain from the Late Nineteenth Century

One striking change relates to transport. The horse population of Britain increased from 1.1 to 3 million during the nineteenth century, peaking in the late Victorian era with a subsequent decline in the twentieth century as steam engines, the internal combustion engine and electrical power replaced horse power. At first glance this could suggest horses as a vector for the disease. However, the *per capita* number of horses actually decreased during the nineteenth century (from approximately 1 horse per 10 people in 1800, to 1 per 13 people in 1900), and the numbers of people and horses in France—a country with a relatively low prevalence of PDB—were almost identical to the numbers in Britain. So the epidemiological data does not fit well with the observed decline in PDB prevalence and severity. If proximity to horses, or indeed any other animals, were implicated then the vector would likely be a zoonosis, but there is no evidence that PDB is an infectious disease: inflammatory markers are not raised, it has not been linked to any microbial vector and it is not transmissible. For these reasons an infectious cause, particularly in relation to horses, seems very unlikely. More plausible is a link to a change in energy consumption.

## Could Domestic Coal Use be an Explanation?

In Britain, the Romans had opened several coalfields by the late second century CE. Coal was used in hypocausts to heat public baths, military forts and the villas of wealthy individuals. After the Romans left Britain (410 CE), there are few records of coal being used until the end of the twelfth century. During the first half of the fourteenth century it began to be used for domestic heating in coal producing areas, though wood was still the preferred fuel. By the middle of the sixteenth century supplies of wood were beginning to fail in Britain and the use of coal as a domestic fuel rapidly expanded [26]. Britain was the first country to experience the industrial revolution and coal was the fuel that drove it. Coal production and the population of Britain both increased enormously during the nineteenth century. Being the first country to undergo an industrial revolution, England’s per capita consumption of fossil fuels was probably ~ 150 times the world average in 1800 and still eleven times the world average by 1900 when other counties had industrialized. By the end of the nineteenth century energy consumption in Britain was fuelled almost entirely by coal [27, 28], and an extensive transport network to deliver coal had been established. Coal prices were lowest in the north-west and north-east of the country, thanks to their proximity to coalfields and lower transport costs [29].

**Fig. 3:**
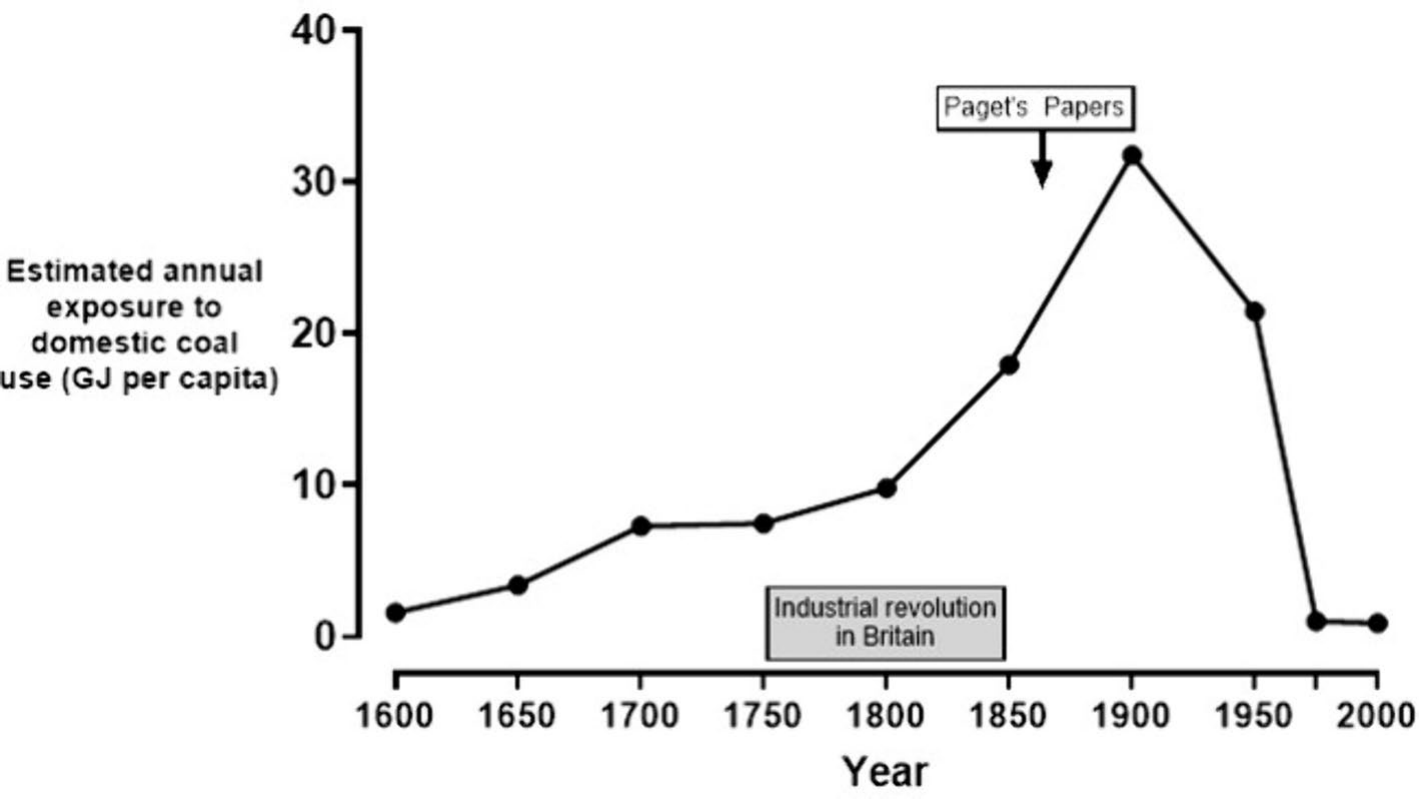
The relationship between estimated exposure to domestic coal burning (gigajoule per capita) in England and Wales from 1600 to 2000. Note the rapid rise during the industrial revolution reaching a peak in the late nineteenth century, around the time Paget first described the disease. There was a rapid fall in the twentieth century as gas and electricity were increasingly used to heat homes and for cooking. Burning coal for domestic purposes was prohibited in Britain in 2023

Coal continues to be burnt in power stations throughout the world to generate electricity, but there has been a marked change in household use of coal. Could patterns of domestic coal use provide an explanation for the rise and fall of Paget’s disease? From changes in coal production [30] and knowledge of population change it is possible to make estimates of exposure to domestic coal in Britain, by factoring in estimates of the proportion of coal production being used for domestic purposes [27, 30, 31] (50% from 1600 to 1700, falling to 22% by 1900, 13% by 1975 and 3% by 2000). Calculated this way, estimated exposure to domestic coal burning rose threefold in the nineteenth century and began to fall after 1900, as domestic energy consumption began to switch away from coal to gas and electricity. Figure 3 illustrates this relationship.

This data fit in well with the cohort mortality data reported by Barker and Gardner [12] (Fig. 2), but does not explain Britain’s peculiarity. By 1905 total coal production in the USA exceeded that of Britain, and Germany was not far behind (351 vs. 236 vs. 174 million short tons, respectively). The use of coal for heating was high in the USA from 1920 through the mid-1940s and then began to decline sharply. At its peak, more than half United States households used coal for home heating, and the majority of these households used bituminous coal, as did those in Britain [32]. However, a crucial difference was that well into the twentieth century, British homes were almost always heated by coal burnt on an open hearth, whereas in Germany closed stoves (*Kachelöfen*) were used. Closed stoves for cooking were introduced into wealthier British homes in the early nineteenth century, but for poorer households, open hearths were used for cooking well into the nineteenth century. In the USA, where Paget’s disease was more prevalent in the colder north-east than the warmer southern states, open hearths were commonly used for burning wood, but closed German-style cast-iron stoves and furnaces for burning coal were common features in American homes after 1865 [33]. In the low prevalence regions of Scandinavia and Ireland, wood and peat, respectively, remained widely used domestic fuels.

Colonization was seen as a solution to overcrowding and poverty, and over 20 million British people emigrated to North America, Australia, South Africa or New Zealand between 1815 and 1914, half of whom left after 1870 [34]. The most common mutation associated with PDB is rs104893941, a missense C > T change causing a P392L change in the p62/SQSTM1 protein. This mutation is indeed more common in European than other populations but the allele frequency is low—0.1 to 0.2% [35]. The colonial settlers included a small proportion carrying, and passing on, *SQSTM1* mutations [36], but this alone cannot explain the relatively high prevalence of Paget’s disease in the settler communities observed in 1970s surveys. Notably, Paget’s disease was reportedly rare in people indigenous to these lands [37]. Settler communities established coal industries in the newly occupied lands and heated their homes in the same way as they had in Britain. In New Zealand and Western Australia, for example, coal mining began in the mid-nineteenth century, with coal being used for domestic heating using open hearths well into the twentieth century. As in British and European centres, with use of coal diminishing in favour of gas and electrical heating there has been a significant fall in the prevalence and severity of PDB in New Zealand [38], and in Western Australia the severity of PDB has decreased significantly in recent decades [39].

## What Could the Factor be?

Paget’s disease is unique to humans and with few parallels to other human diseases. Authorities from Fuller Albright onwards have been notably reticent to speculate as to the nature of the disease, but in 1974 Rasmussen and Bordier suggested that it could be viewed as a benign focal neoplastic disorder [40]. A number of points support this view. Although the clinical manifestations are osteoclast-driven, anti-osteoclastic treatment with potent bisphosphonates does not permanently cure the disease, suggesting that a ‘stem cell’ (possibly of non-osteoclastic origin) remains within bone. When PDB relapses after bisphosphonate treatment, the rise in ALP, reflecting the ‘active pagetic mass’, follows the Gompertz curve, a common mathematical description of tumor growth [41]. The transmission of PDB by bone grafting is also in keeping with a quasi-neoplastic process [42]. A perennial problem in explaining PDB is the apparently synchronous but random distribution of lesions within the skeleton. A parallel here might be metastatic disease by hematogenous spread. This would fit in with the random distribution of lesions, but new pagetic foci do not develop after initial diagnosis [43], suggesting just a single episode of hematogenous spread has occurred—obviously atypical of metastatic disease in general. An active focus can occasionally undergo malignant transformation, as Paget described [10], presumably because of somatic mutation, but pagetic osteosarcoma shows the same declining incidence and increasing age at presentation as PDB [44].

If both the domestic coal exposure and the quasi-neoplasia hypotheses are correct, then it implies there is (or was) a coal-related factor responsible. Indeed, coal contains a number of polycyclic aromatic hydrocarbons [45] and also non-organic elements, including nickel, vanadium, chromium, cobalt, arsenic, cadmium, molybdenum, lead, thallium and antimony [46], with the potential to induce neoplasia. How these compounds or elements could cause PDB is a matter for future research. Cadmium may offer one example: the SQSTM1/p62 protein which plays an important role in transporting polyubiquinated-proteins to the proteasome and autophagosomes for degradation is thought to have a key role in the pathogenesis of PDB [1, 24, 41]. Cadmium has been shown in vitro to increase the levels of polyubiquinated-proteins and induce autophagy in macrophage/monocyte cells [47].

Coal comes in different forms. Bituminous coal, which was the main form used for domestic heating and cooking in Britain, contains more volatile matter (14–46%) than anthracite (3–14%). Coal from different regions has different degrees of volatile matter and different non-carbon elements—for example, the cadmium, thallium, arsenic and lead content of coal ash can vary tenfold [48]—so this could also be a factor in within- and between-country variation in PDB prevalence.

## Conclusions

Paget’s disease is unique to humans, and as our biology is not that different to other mammals, it is reasonable to infer that it is linked to some uniquely human activity. Its rise in prevalence and severity in the nineteenth century and subsequent decline in the twentieth century seem indisputable. Its distinctive geographic distribution and temporal change offer clues as to its etiology. The data linking the rise and fall of domestic coal use and the rise and fall of PDB prevalence and severity is plausible, particularly in the British context, although the evidence necessarily remains circumstantial. The coal hypothesis does not exclude the possibility that other factors could be important. In Quebec, which has mines producing niobium, copper, titanium, gold, zinc, copper, silver and graphite, but not coal, PDB has been linked to both mining and to exposure to wood-burning fires [49], though in Quebec too the prevalence is declining [7]. Invoking such an environmental trigger does not preclude the possibility that PDB could emerge without that trigger, particularly when there is a strong genetic driver. Indeed, as sporadic PDB becomes rarer, genetically driven forms may appear relatively more common. The coal hypothesis raises as yet unanswerable questions: Is age at exposure or duration of exposure important, as suggested by the lower rates of PDB in people of British descent who were born in Western Australia compared to those who had migrated there as adults [50]? Is the severity of the disease at presentation the result of a cumulative dose effect? If PDB is indeed a quasi-neoplastic disorder, can somatic mutations be identified in pagetic bone cells that are not present in cells from uninvolved bone?

The cause of Paget’s disease remains an enigma and the hypothesis outlined in this paper, suggesting a link to rapid industrialization and implicating domestic coal use, is probably untestable at the population level, given the worldwide change in coal usage. However, this hypothesis could stimulate further research into the biology of this unique disease.
